# Pornography cyberaddiction and impulsivity among medical tunisian students

**DOI:** 10.1192/j.eurpsy.2021.1542

**Published:** 2021-08-13

**Authors:** N. Meseddi, M. Bouhamed, O. Bouattour, A. Chamseddine, F. Charfeddine, L. Aribi, J. Aloulou

**Affiliations:** Psychiatry, Hedi chaker hospital, Sfax, Tunisia

**Keywords:** pornography, Cyberaddiction, Impulsivity, Medical Students

## Abstract

**Introduction:**

Medical studies have always been considered as very stressful. Although these students are generally among the most academically successful students, they are not spared from developing both substance and behavioral addictions, particularly pornography cyberaddiction.

**Objectives:**

To evaluate the pornography cyberaddiction in a group of tunisian students, to study their impulsivity and determinate the link between these two entities.

**Methods:**

A descriptive and analytical cross-sectional study including 155 medical students. We used the S-IAT sex : to evaluate pornography cyberaddiction and Barrat bis 10 : to evaluate impulsivity.

**Results:**

The average age of students was 25.8 ±3.5 years old. The sex ratio (M/W) was 0,72. This medical students were single in 76.8%, had a high socio-economic status in 99.4% of case. They had a personal psychiatric history in 15.0%. They are smokers in 20%, consume alcohol in 30.3% and cannabis in 9% of case. The mean score of : the BIS 10 was 63.3 and the S-IAT was 15.6. The viewing of pornographic movies started around the age of 15 years old. Pornographic sites are the most frequently used tool (58.2%). The factors correlated with this addiction are: male sex (p=0.014), tobacco consumption (p=0.012), alcohol consumption (p=0.02) and impulsivity (p=0.03).

**Conclusions:**

It resort from our study that medical students may suffer from pornography cyberaddiction. This increased use may be associated with impulsivity and substance use. Psychological support aimed specifically should be used to protect medical students.

